# The Impact of Radiation-Induced DNA Damage on cGAS-STING-Mediated Immune Responses to Cancer

**DOI:** 10.3390/ijms21228877

**Published:** 2020-11-23

**Authors:** Quinn Storozynsky, Mary M. Hitt

**Affiliations:** Department of Oncology, University of Alberta, Edmonton, AB T6G 2E1, Canada; storozyn@ualberta.ca

**Keywords:** radiation, cancer, DNA damage, nucleic acid sensing, cGAS–STING signaling, type I interferon, antitumor immunity

## Abstract

Radiotherapy is a major modality used to combat a wide range of cancers. Classical radiobiology principles categorize ionizing radiation (IR) as a direct cytocidal therapeutic agent against cancer; however, there is an emerging appreciation for additional antitumor immune responses generated by this modality. A more nuanced understanding of the immunological pathways induced by radiation could inform optimal therapeutic combinations to harness radiation-induced antitumor immunity and improve treatment outcomes of cancers refractory to current radiotherapy regimens. Here, we summarize how radiation-induced DNA damage leads to the activation of a cytosolic DNA sensing pathway mediated by cyclic GMP-AMP (cGAMP) synthase (cGAS) and stimulator of interferon genes (STING). The activation of cGAS–STING initiates innate immune signaling that facilitates adaptive immune responses to destroy cancer. In this way, cGAS–STING signaling bridges the DNA damaging capacity of IR with the activation of CD8+ cytotoxic T cell-mediated destruction of cancer—highlighting a molecular pathway radiotherapy can exploit to induce antitumor immune responses. In the context of radiotherapy, we further report on factors that enhance or inhibit cGAS–STING signaling, deleterious effects associated with cGAS–STING activation, and promising therapeutic candidates being investigated in combination with IR to bolster immune activation through engaging STING-signaling. A clearer understanding of how IR activates cGAS–STING signaling will inform immune-based treatment strategies to maximize the antitumor efficacy of radiotherapy, improving therapeutic outcomes.

## 1. Introduction

Ionizing radiation (IR) is a staple therapeutic modality for treating a variety of cancers [1]. IR generates lethal double-strand breaks directly in the DNA of irradiated cells, as well as indirectly through the production of reactive oxygen species that induce breaks. If left unrepaired, DNA-damaged cells may undergo a variety of cellular death modalities including apoptosis, mitotic catastrophe, and autophagy (among others) or enter a growth-arrested state termed cellular senescence [2]. In addition to producing lethal DNA damage that kills cancer cells, IR has emerged as a potent tool for facilitating antitumor immune responses [3]. Some of the pro-immunogenic effects of IR observed in preclinical settings include increasing susceptibility of cancer cells to cytotoxic T cell killing [4,5,6,7], augmenting antigen processing and inducing expression of unique radiation-associated peptides in cancer cells [8,9], inducing irradiated cancer cells to release or express immunogenic molecules that can enhance anticancer immune responses [8,10], and favorably modulating the tumor microenvironment (TME) for immune-mediated antitumor effects [11,12,13,14]. In clinical settings, radiotherapy is currently being explored in combination with a plethora of immune-based therapeutic approaches to optimize antitumor immunity [15,16].

An obvious advantage of treatment modalities or combination therapies that induce antitumor immunity is the potential for systemic eradication of the disease. Following radiotherapy, systemic immune-mediated anticancer effects (often termed abscopal effects) have been reported; though, this effect is rare, and the underlying immunological mechanisms are inadequately characterized [17]. Therefore, to harness the maximum therapeutic potential of patient’s immune systems, a better understanding of the immunology at play following radiotherapy is warranted. It has been suggested that modalities capable of engaging the innate immune system may optimize the therapeutic efficacy of radiotherapy [15]. Cytoplasmic nucleic acid sensing mechanisms represent one component of innate immunity that have been implicated in inducing downstream CD8+ T cell-mediated cancer cell killing following radiation [18]. Specifically, the cGAS–STING pathway has gained enormous attention recently and the activity of this pathway is paramount for optimal antitumor immune activation following IR in preclinical settings (Table 1).

The objective of this review was to highlight how radiation-induced DNA damage leads to activation of innate immune signaling and subsequent CD8+ T cell-mediated tumor destruction through nucleic acid sensing mechanisms involving the cGAS–STING pathway. Understanding the mechanisms involved in producing immune responses against cancer following radiotherapy, as well as how activation or inhibition of key players involved in cGAS–STING signaling affects antitumor efficacy, will aid in informing strategies to improve radiotherapy.

## 2. The Role of cGAS, STING, and Type I Interferons in Antitumor Immunity

### 2.1. A Primer on Immune-Mediated Destruction of Cancer

The immune system can be broadly categorized into two fundamental components called innate and adaptive immunity that coordinate the clearance of pathogens and malignant cells. Some major distinctions between these categories are the differences in specificity and activation-time; innate immunity involves fast-acting non-specific defense mechanisms, whereas adaptive immunity develops much slower and involves highly specific targeting of threats.

A crucial component of innate immunity involves the sensing and communication of threats to the adaptive immune system. Molecular motifs, termed pathogen-associated molecular patterns or damage-associated molecular patterns (DAMPs), are detected by pattern recognition receptors of innate immune cells, initiating production of various cytokines or chemokines that function to alert and activate further immune responses [31]. In this way, innate immunity produces early inflammatory events, shaping the intensity and extent of subsequent immune responses.

An important component of the adaptive immune response is the evolutionarily selected class of potent immunological effectors, termed CD8+ cytotoxic T cells, that systemically and specifically destroy intracellular pathogens or cancerous cells. All nucleated cells that compose our bodies are decorated with specialized antigen presenting complexes on the cell-surface termed major histocompatibility complex class I molecules (MHC I, or human leukocyte antigen I in humans) that serve as signaling devices to our immune system [32]. CD8+ cytotoxic T cells engage infected or transformed cells by recognizing foreign peptide fragments (i.e., peptides produced by intracellular degradation of proteins that are microbial-derived, of different tissue origin, or mutated) bound to MHC I on the target cell surface [32]. When the T cell receptor (TCR) on the surface of a CD8+ T cell possesses specific affinity to a peptide–MHC I complex on the surface of the target cell, the T cell will directly kill the cellular target by triggering apoptosis through release of granzyme B and perforin, or death receptor signaling (i.e., Fas/FasL) [33]. In this way, cells residing in our body harboring harmful infectious agents or that have initiated tumorigenesis display antigens indicative of a compromised cellular state and can be selectively eliminated by the immune system to preserve normal host function.

Dendritic cells (DCs) represent the cellular bridge connecting innate and adaptive arms of the immune system. DCs sense innate danger signals and cues from the microenvironment, process and present antigens, and provide costimulatory molecular signals to activate adaptive immune effectors—ultimately initiating an adaptive immune response against foreign (non-self) entities [34]. In the context of antitumor immunity, DC subtypes specialized at cross-presenting tumor-antigens (i.e., conventional DCs type 1; cDC1s) to tumor-reactive cytotoxic CD8+ T cells are the most important [35]. Cross-presentation is a mechanism in which DCs process and present extracellular antigens on MHC I molecules to CD8+ cytotoxic T cells (that is, instead of on MHC class II molecules to CD4+ T helper cells). CD8α+ DCs are a subset of conventional DCs that require the Batf3 transcription factor (in mice) for development and exhibit efficient cross-presentation of viral and tumor antigens enabling CD8+ T cell-mediated defense [35,36].

Optimal immune-mediated tumor destruction requires not only the CD8α+ DC lineage but also type I interferon (IFN) signaling. Type I IFNs are immunomodulatory factors that mobilize host defenses to counter viral and bacterial pathogens [37], as well as to destroy cancer cells [38,39]. They include IFNα (comprising 13 subtypes), IFNβ, and the other less-characterized IFNε, IFNκ, and IFNω, all of which are secreted and act on a common IFNα/β receptor (IFNAR) that is present on all nucleated cells. Binding of type I IFNs to this receptor activates multiple signal transduction pathways inducing diverse responses, including antiviral and antiproliferative activities [40]. Type I IFNs can serve as elements bridging innate and adaptive immune responses by facilitating DC maturation, increasing DC costimulatory molecule expression, and enhancing DC lymph-node migratory capacity—each of which amplifies DC-mediated stimulation of T cells [38,41]. Studies using highly immunogenic tumor models capable of producing spontaneous antitumor T cell activity revealed that type I IFN is essential for regulating the capacity of CD8α+ DCs to prime CD8+ T cells and facilitate subsequent immune-mediated antitumor responses [42,43]. Type I IFN responsiveness of DCs is also required for induction of tumor-antigen specific CD8+ T cells [44].

Collectively, these data validate the essential roles of type I IFNs, cross-presenting DCs, and CD8+ T cells for maximizing anticancer activity.

### 2.2. Nucleic Acid Sensing and Sources of Cytosolic DNA

Under normal cellular homeostatic conditions, the nucleus contains the genome while the cytosol remains relatively free of double-stranded DNA (dsDNA). Thus, aberrant levels of dsDNA within the cytosol may indicate pathogenic threats or compromised cellular states that could threaten host homeostasis. In this context, molecular machinery capable of detecting and communicating potential breaches in homeostasis becomes paramount for preserving normal host function. Recent studies have characterized a vital nucleic acid sensing pathway directing type I IFN production [25,29,45,46,47,48,49,50]. Key mediators within this pathway are cyclic GMP-AMP (cGAMP) synthase (cGAS) and stimulator of interferon genes (STING). When dsDNA is present in the cytosol, the cGAS–STING pathway induces innate immune signaling which includes production of type I IFNs and other co-regulated cytokines [45,46,49,50,51]. Intriguingly, the cGAS–STING axis has been suggested to have emerged early in the evolutionary history of animals, likely predating the establishment of IFN-mediated innate immune signaling [52].

Cytosolic dsDNA (the ligand for cGAS–STING activation) can emerge in several contexts from both foreign and host sources. DNA species derived from microbial pathogens such as viruses and bacteria can be sources of cytosolic dsDNA that trigger the cGAS-STING-type I IFN axis [53]. Self- (or host) DNA can also accumulate in the cytosol following DNA damage in normal and malignant cells, triggering cGAS–STING-mediated signaling [19,20,25]. Furthermore, following DNA damage, fragmented chromatin may be released into the cytoplasm during mitotic slippage (which is the reset of interphase from spindle checkpoint arrest without cellular division) [54]. Interestingly, DNA damaging agents, including IR, can increase cellular resistance to virus and bacteria challenge via induction of STING signaling and subsequent priming of innate immune responses [29]. Furthermore, cGAS–STING signaling is activated within DCs that have internalized genetic material originating from tumor cells into the cytosol [27,50]. cGAS–STING signaling can also be pathogenic, as activation of this pathway by cytosolic self-DNA accumulation (due to dysfunctional DNase activity) is believed to cause autoimmune disorders such as Aicardi–Goutieres syndrome [55,56].

Overall, the cGAS–STING pathway is an important transduction circuit for activating immunological defenses against various pathogens and malignancies, but is also implicated in exacerbating autoimmune responses through dysregulated self-DNA accumulation.

### 2.3. cGAS, STING, and Type I Interferon Signaling Transduction

cGAS is a cytosolic dsDNA sensor; when bound to dsDNA, the nucleotidyl transferase activity of cGAS is stimulated initiating a signaling cascade involving STING, leading to production of type I IFNs [49]. Unbound cGAS exists in an autoinhibited conformation [57]. Upon dsDNA binding, conformational changes expose the catalytic pocket of cGAS [58]. Steric interactions between the activation loop of cGAS and bound dsDNA alter the position of the activation loop, leading to rearrangement of the catalytic site [57]. In this catalytically competent conformation, cGAS produces two phosphodiester linkages in a stepwise manner between GTP and ATP molecules resulting in the synthesis of a second messenger molecule, 2′,3′-cGAMP [58,59,60].

2′,3′-cGAMP is a noncanonical cyclic dinucleotide (CDN) containing mixed 2′-5′ and 3′-5′ phosphodiester bonds (hence, the denotation, 2′,3′-cGAMP) [58,59,60]. CDN second messenger molecules of prokaryotic origin contain two 3′-5′ phosphodiester bonds (denoted as 3′,3′-CDNs) and are involved in regulating a variety of bacterial functions [61,62]. It is important to note that the 2′-5′ phosphodiester bond of 2′,3′-cGAMP is distinct from the well-characterized bacterial derived 3′,3′-CDNs. It has been recently revealed that STING is bound more tightly by cGAS produced 2′,3′-cGAMP than by bacteria-derived 3′,3′-CDNs [58,59,60,63], revealing a previously unappreciated role for a mammalian-derived, endogenously produced CDN (i.e., 2′,3′-cGAMP) in innate immune signaling. In addition to high-affinity binding and activation of STING, 2′,3′-cGAMP functions as a messenger warning nearby unharmed cells of pathogenic threat, either through translocation of the CDN through gap junctions to neighboring cells [64] or through infection of neighboring cells with newly assembled virions that have co-packaged the CDN during assembly [65]. In these ways, 2′,3′-cGAMP also functions as a paracrine signaling molecule for STING activation and innate immune priming of cells ignorant of impending pathogenic threats. In summary, cGAS functions as a translator communicating the presence of cytosolic dsDNA into a language (i.e., 2′,3′-cGAMP) STING can understand.

STING is an endoplasmic-reticulum-resident protein that dimerizes to fulfill its role in signaling type I IFN expression [45,47]. STING is essential for production of type I IFNs following virus infections, intracellular DNA exposure, and for protection against herpes simplex virus type-1 infection in mice [45,46]. cGAMP binding induces conformational changes in STING [63] and leads to oligomerization of STING and TANK-binding kinase 1 (TBK1) [66], which may be facilitated by polyubiquitination of STING [67]. The resultant STING–TBK1 complex appears to enable TBK1 phosphorylation of STING [66]. Next, phosphorylated STING recruits interferon regulatory factor 3 (IRF3) and serves as an adaptor molecule facilitating phosphorylation of IRF3 by TBK1 [68,69]. Phosphorylated IRF3 dimerizes and translocates to the nucleus where it acts as a critical transcription factor for expression of type I IFNs and other co-regulated genes [70]. Additionally, STING activates NF-κB, another central pro-inflammatory signaling transcription factor [51]. To prevent sustained activation of STING-mediated innate immune responses, STING is negatively regulated through inhibitory phosphorylation [71], ubiquitin-mediated degradation [72], and trafficking-mediated degradation by lysosomes [73].

In summary, the cGAS–STING pathway enables initiation of innate immune responses through detection of cytosolic dsDNA and subsequent induction of type I IFNs.

## 3. The Connection between Radiation-Induced DNA Damage and Innate Immune Activation

Damage to the genetic code threatens the survival of eukaryotic cells and if not rectified can lead to deleterious tumorigenic processes. In this respect, mechanisms that repair DNA damage or facilitate elimination of damaged cells are critical for maintaining integrity of the host unit. Immune-mediated processes enable surveillance of malignant cells [74] and activation of cGAS–STING associated inflammation may help facilitate surveillance activity, serving as a barrier to tumorigenesis [75]. Indeed, the cGAS–STING pathway is associated with promoting antitumor immune responses [26,50,76], although it should be noted the same pathway can paradoxically facilitate tumor progression in particular contexts [75]. IR is a potent DNA damaging agent and was classically characterized as a direct cytocidal therapeutic modality. However, there is increasing appreciation for IR as an inducer of antitumor immune responses, including via activation of cGAS-STING-type I IFN signaling [25,26,28].

How is it that DNA damage leads to activation of innate immune pathways? Early reports highlighted the role that DNA damage or DNA damage associated proteins (i.e., DNA-dependent protein kinase) play in activating components of innate immune signaling (i.e., IRF3) [77,78]. It has since been revealed that DNA damage, including damage due to IR, leads to the formation within the cytoplasm of micronuclei that contain chromatin/dsDNA [19,20]. Micronuclei form when DNA species (such as fragmented or whole chromosomes/chromatids) fail to incorporate in daughter nuclei post-mitosis due to improper spindle attachment and segregation during anaphase [79]. In the cytosol, the nuclear envelope of micronuclei can breakdown and rupture, providing the dsDNA substrate necessary for cGAS activation and subsequent innate immune signaling [19,20]. It has been proposed that cell-cycle progression is a prerequisite for innate immune activation mediated by micronuclei [19,20]. Consistent with this notion, the use of cell-cycle checkpoint inhibition to drive proliferation in concert with IR treatment increased micronuclei formation in vitro and improved in vivo efficacy [80].

However, proliferation is not a prerequisite for all innate immune signaling activators. DNA damage (including radiation-induced) is known to induce cellular senescence [21,24,81], a state in which cells remain metabolically active but no longer proliferate (note: in some cases senescent cells may “escape” senescence [82,83]). Although senescent cells do not progress through the cell-cycle, the release of cytoplasmic chromatin fragments (CCFs) from the nucleus due to nuclear lamin B1 degradation appears to trigger cGAS and innate inflammatory responses thereafter [21,24]. Additionally, mitochondria DNA (mtDNA) may also be a source for stimulating innate immune responses through cGAS–STING [22,23,84,85]. Oxidized mtDNA derived from irradiated cancer cells used as vaccines activated STING signaling in DCs which was critical for eliciting antitumor immune responses in preclinical models [23]. Furthermore, super resolution imaging revealed that mtDNA was released into the cytosol following irradiation of tumor cells and may play a vital role in innate immune activation [22].

**Figure 1:**
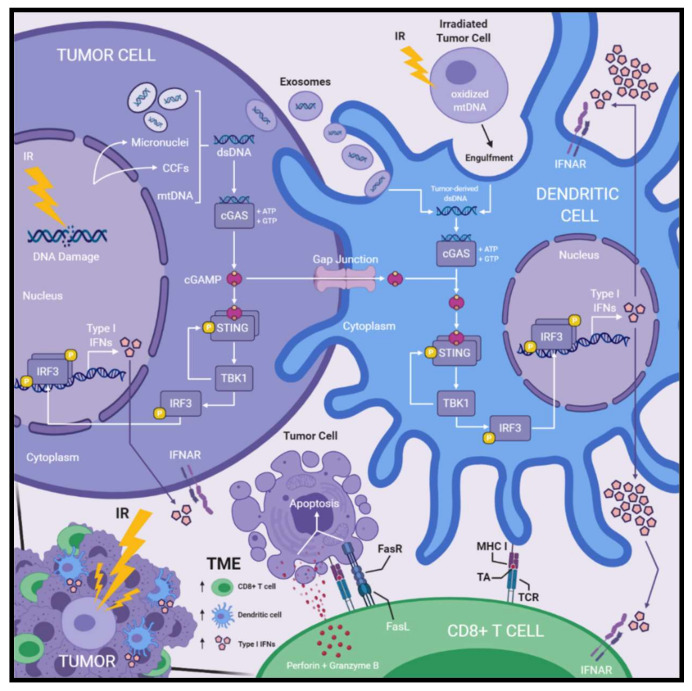
Radiation-induced DNA damage activates cGAS–STING signaling, type I interferon production, and immune activation within the tumor microenvironment. Radiation-induced DNA damage leads to accumulation of cytosolic dsDNA within irradiated tumor cells. Micronuclei, CCFs, and mtDNA have been reported as sources of cytosolic dsDNA. cGAS detects cytosolic dsDNA and produces cGAMP, a secondary messenger molecule formed by phosphodiester linkages between ATP and GTP molecules. cGAMP binding of STING induces conformational changes that enable the recruitment of TBK1, which then phosphorylates STING. Phosphorylated STING recruits IRF3, which is next phosphorylated by TBK1. Phosphorylated IRF3 dimerizes and translocates to the nucleus where it functions as a transcription factor for the expression of type I IFNs. Following irradiation of tumor cells, DCs acquire tumor-derived dsDNA within the cytosol via internalizing tumor-derived exosomes or engulfment of irradiated tumor cells. Furthermore, cGAMP produced within irradiated tumor cells is transferred to the cytosol of DCs via gap junctions. Tumor-derived dsDNA or cGAMP activates cGAS–STING signaling and subsequent production type I IFNs within DCs as described above. DCs produce greater amounts type I IFNs compared to other cellular compartments in the TME. Type I IFNs are secreted and act on IFNAR receptors initiating a variety of responses including induction of IFN-stimulated genes, DC activation and maturation, as well as CD8+ T cell activation. Activated DCs cross-present TA on MHC I molecules which is recognized by tumor-reactive CD8+ T cells via their TCR. Activated CD8+ cytotoxic T cells recognize TA–MHC I complexes presented on the surface of target tumor cells and induce apoptosis through the release of perforin and granzyme B, or death receptor signaling involving FasL/FasR. Treatment with IR leads to the accumulation of TA-specific CD8+ T cells, specialized cross-presenting DCs, and type I IFNs within the TME—all of which are paramount for optimal antitumor immune responses induced by radiotherapy. Abbreviations: cytoplasmic chromatin fragments (CCFs); dendritic cells (DCs); double-stranded DNA (dsDNA); interferon (IFN); IFN-α/β-receptor (IFNAR); ionizing radiation (IR); mitochondrial DNA (mtDNA); T cell receptor (TCR); tumor-antigen (TA); tumor microenvironment (TME).

Collectively, cGAS–STING signaling and subsequent innate immune activation following DNA damage may function to alert the immune system to the presence of aberrant cellular phenotypes with potential for neoplastic transformation. Thus, radiation-induced DNA damage may permit exploitation of this innate immune activating pathway via promoting cytosolic dsDNA accumulation and enable improved therapeutic efficacy against cancer (Figure 1).

## 4. Radiation-Induced Antitumor Responses Involving cGas, STING, or Type I Interferons

### 4.1. Radiation-Induced Immunological Contributions to Antitumor Immunity

The antitumor efficacy of radiotherapy depends on the interconnected activities of type I IFNs, cross-presenting DCs, and CD8+ cytotoxic T cells for maximum therapeutic potential, implicating IR as a genuine activator of antitumor immunity [12,13,25,86,87,88].

Type I IFN responsiveness is indispensable for optimal antitumor immunity following IR treatment. The efficacy of IR was abolished in B16F10 melanoma and EL4 lymphoma models established in IFNAR-deficient mice while showing robust tumor control in wild-type mice, indicating the necessity for host type I IFN responsiveness to produce optimal radiation-mediated antitumor activity [86]. The efficacy of IR in combination with anti-CTLA4 immune checkpoint blockade was also abrogated in IFNAR1-deficient mice [25]. Increases of IFNβ in the TME due to IR was crucial for enhancing DC stimulation of tumor-antigen-specific CD8+ T cells [86]. Furthermore, type I IFN signaling was critical for accumulation of tumor-infiltrating CD8+ and CD4+ T cells, DCs, and macrophages following irradiation of B16 melanoma models [88]. Recent data indicate that the activation status and cytolytic activity of tumor-associated T cells following IR treatment also depends on intact type I IFN signaling [88]. Together, radiation-induced type I IFN appears vital for enhancing DC priming of T cell-mediated antitumor activity [25,86], recruitment of tumor-infiltrating immune cells [88], and direct enhancement of T cell activation and cytolytic function [88].

DCs are crucial for mediating antitumor immune responses following IR treatments. Lee et al. (2009) observed that IR increased DC maturation and presentation of tumor-antigen [12]. In B16gp melanoma models (B16F10 cells that express lymphocytic choriomeningitis virus-derived gp), IR increased expression of CD70 and CD86 costimulatory molecules on DCs and increased the number of tumor-antigen specific CD8+ T cells in the TME, suggesting that IR facilitated DC maturation and cross-priming capabilities towards effector T cells [87]. Intriguingly, selective depletion of DCs or CD70 costimulatory molecule blockade, reduced the therapeutic efficacy of IR, delineating the importance of DCs and cross-priming for radiation-induced antitumor immunity [87]. Furthermore, the antitumor efficacy of IR towards CT26 colon cancer models was greatly compromised in mice deficient for CD8α+ DCs, indicating the necessity for specialized cross-presenting DC subtypes for ideal tumor control using IR [13]. Vanpouille-Box et al. (2017) observed a marked increase in tumor-infiltrating CD8α+ DCs and CD70 costimulatory molecule surface expression following hypofractionated IR schedules [25]. The efficacy of IR in combination with anti-CTLA4 immune checkpoint blockade was also abrogated in mice deficient for CD8α+ DCs [25]. Lastly, Blair et al. (2020) demonstrated that cDC1 activation is vital for mediating radiation-induced antitumor immunity [89]. Following treatment with IR, radioimmunogenic tumor models (i.e., defined as models exhibiting CD8+ T cell-*dependent* antitumor efficacy following IR) showed activation of cDC1s, whereas poorly radioimmunogenic tumor models (i.e., defined as models exhibiting CD8+ T cell-*independent* antitumor efficacy following IR) failed to do so [89]. This finding suggests activation of cDC1 may be important for CD8+ T cell-mediated tumor destruction following IR. Indeed, when the authors administered an adjuvant (i.e., poly I:C) to activate cDC1s in poorly radioimmunogenic tumor models, they observed greatly enhanced therapeutic efficacy in concert with IR in a manner dependent on cDC1s and CD8+ T cells [89].

The role of CD8+ T cells in radiation-induced immune responses against cancer is also paramount. Depletion of CD8+ T cells abrogated the therapeutic efficacy of IR, but not depletion of CD4+ T cells or macrophages highlighting the critical requirement for CD8+ T cells in facilitating radiation-induced tumor control [87]. Interestingly, IR maintained T cell activation for longer time periods within the TME compared to unirradiated controls [87]. CD8+ T cells were also deemed essential for the efficacy of IR in B16 melanoma models [12] and IR alone increased the amount of tumor-antigen specific CD8+ T cells in both irradiated and unirradiated areas of B16-OVA melanoma lung metastasis models [90].

The aforementioned studies indicate type I IFNs, specialized cross-presenting DCs, and CD8+ cytotoxic T cells are paramount for radiation-induced antitumor immune responses, but what is the link between IR and antitumor immunity?

### 4.2. Activation of cGAS–STING and Type I Interferon Production Following Ionizing Radiation Treatment

Recent data implicate activation of the cGAS–STING pathway in DCs as critical for type I IFN production in the TME and the efficacy of radiotherapy in mouse tumor models [26]. Post-IR treatment, CD8+ T cells taken from draining lymph nodes of STING-deficient mice were impaired, although function was restored by intratumoral administration of exogeneous IFNβ. A role for DCs in STING-dependent T cell activation was suggested by experiments showing that STING-deficient DCs exposed to irradiated tumor cells in vitro were unable to produce IFNβ or cross-prime CD8+ T cells, whereas wild-type DCs did both. Similar results were obtained using cGAS-deficient DCs, suggesting cGAS-sensing of tumor-derived dsDNA within DCs was involved and potentially important for mediating the capacity of DCs to stimulate CD8+ T cells. Lastly, the efficacy of IR was reduced when tumors were established in mice with IFNAR1-deficient DCs. These findings indicate that cGAS–STING signaling, type I IFN production, and intact type I IFN responsiveness of DCs are important for optimal efficacy following IR.

In contrast, other studies have indicated that tumor cells themselves, as opposed to the hematopoietic compartment (i.e., DCs [26] or myeloid cells [86]), could be essential sources for type I IFNs in the TME post-IR treatments [25]. Knockdown of cGAS or STING in multiple breast cancer cell lines severely compromised IFNβ release in response to IR in vitro and reduced systemic tumor control of bilateral flank models following IR and anti-CTLA4 treatment, demonstrating the importance of intact cGAS-STING-type I IFN signaling in tumor cells for optimal efficacy [25]. Lastly, investigations using a STING agonist to induce antitumor immunity in the absence of IR found that endothelial cells of the vasculature in the TME were principle producers of type I IFN, highlighting the role of yet another cellular compartment for optimal antitumor responses [91]. However, the role endothelial cells play in type I IFN production following IR remains to be determined.

These studies indicate that the cGAS–STING pathway and type I IFN signaling are central in coordinating antitumor immune responses involving DC priming of CD8+ T cells following IR. How is cGAS–STING and subsequent production of type I IFNs being triggered in DCs following radiotherapy?

### 4.3. Sources Activating cGAS–STING Signaling in Dendritic Cells

Several studies have highlighted possible mechanisms responsible for DC acquisition of tumor-derived dsDNA. One process of cell-to-cell communication involves secretion of cargo-containing exosomes (i.e., small membrane-bound microvesicles) [92]. Exosomes carrying tumor-derived dsDNA from irradiated breast cancer cells increased expression of costimulatory molecules CD40, CD80, CD86 and initiated type I IFN production in DCs in a STING-dependent manner [27]. DCs were shown to internalize exosomes derived from irradiated breast cancer cells in vivo leading to activation of tumor-specific CD8+ T cells [27]. These data indicate that following IR, exosomes may mediate transfer of tumor-derived dsDNA to DCs for initiation of downstream immune responses. Other studies support a similar mechanism. Exosomes containing DNA from cancer cells treated with a topoisomerase I inhibitor were found to activate DCs in a STING-dependent manner [93], indicating that exosome-mediated transfer of tumor-derived dsDNA to DCs may be a general effect of DNA damaging agents.

STING-mediated induction of type I IFNs and subsequent antitumor immune attack has also been suggested to be initiated in DCs via direct uptake of tumor-derived DNA into the cytosol [50]. Studies by Fang et al. (2020) support this mechanism in the context of radiotherapy [23]. Following engulfment by DCs, irradiated cancer cells deposited oxidized tumor mtDNA in the cytosol thus activating DC STING signaling which was critical for eliciting antitumor immune effects in preclinical models [23].

Finally, other studies indicate that DC uptake of tumor-derived dsDNA may not be required at all for activating type I IFN production and subsequent antitumor immune responses, but rather tumor-derived cGAMP may be a crucial mediator. cGAS was dispensable in host cells but not cancer cells for optimal antitumor responses, suggesting tumor-derived cGAMP downstream of dsDNA sensing may be mediating activation of innate immune cells rather than dsDNA itself [94]. Schadt et al. (2019) revealed that cancer cells transfer cGAMP through gap junctions to DCs, activating DC-mediated type I IFN production in a host-STING-dependent manner [28]. In a radiotherapy context, efficacy was abrogated in CT26 colon cancer models using cGAS-deficient tumor cells, indicating cancer-cell-intrinsic expression of cGAS is required for the efficacy of IR [28]. Further, cGAS was dispensable in DCs but not cancer cells for optimal CD8+ T cell-mediated cancer immunosurveillance, indicating DC activation was not occurring via nucleic acid sensing [28]. Irradiated cancer cells increased production of extracellular cGAMP in vitro and were dominant producers of extracellular cGAMP in vivo (as opposed to host cells) [30]. Lastly, both extracellular cGAMP and host-STING were required for optimal efficacy of IR and cGAMP depletion reduced tumor-infiltrating DCs and CD8+ T cell activation [30]. Together, these findings indicate that cancer-cell-derived cGAMP, as opposed to cancer-cell-derived dsDNA, is sensed by host-STING initiating antitumor immune responses following IR treatment.

Overall, studies investigating the capacity of IR to produce antitumor immune responses suggest radiotherapy facilitates transfer of tumor-derived dsDNA or cGAMP to the cytosol of DCs, which then activates cGAS–STING signaling and production of type I IFNs, ultimately enhancing DC priming capabilities and subsequent CD8+ T cell-mediated antitumor activity (Figure 1).

## 5. STING-Independent Activation of Type I Interferons Following Radiation

Previous studies have reported that RNA sensing pathways (i.e., mitochondrial antiviral-signaling protein (MAVS)-mediated), Toll-like receptor pathways (i.e., Myd88-, TRIF-, TLR4-, or TLR9-mediated), or DAMP sensing pathways (i.e., extracellular-ATP-mediated) were unnecessary for inducing antitumor CD8+ T cell responses, but STING was required, strongly suggesting that innate immune sensing of cancer is chiefly mediated by STING signaling [50]. Although STING plays a critical role, it is not the whole story when it comes to radiation-induced type I IFNs and later immune activation (Table 2). Like STING, MAVS is an adaptor molecule occupying a central position in production of type I IFNs. Interestingly, MAVS signaling is initiated by cytosolic RNA detection as opposed to DNA detection [95].

**Figure 2:**
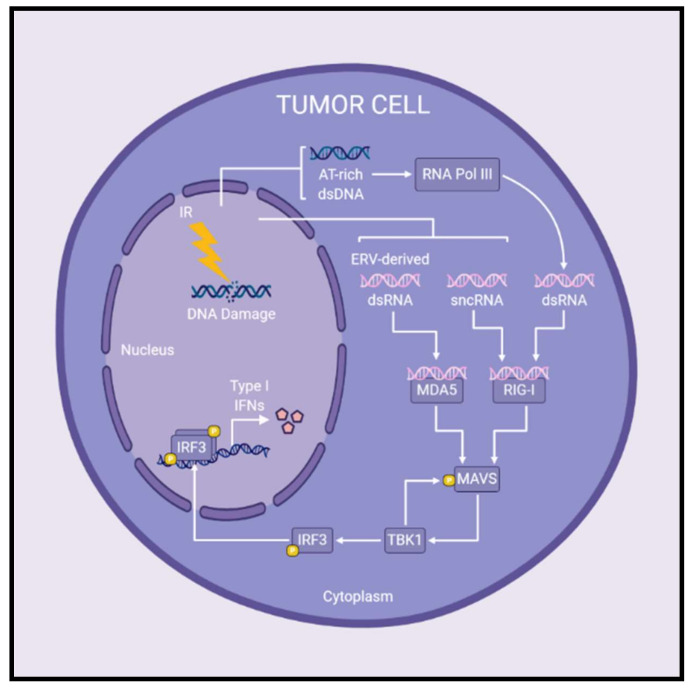
Radiation-induced MAVS signaling leads to the production of type I interferons in cancer cells. Following IR treatments, cytosolic RNA species have been reported to activate MAVS signaling and subsequent production of type I IFNs. MDA5 and RIG-I are cytosolic RNA sensors upstream of the adaptor protein, MAVS. Upon detection of cytosolic RNA, MDA5 and/or RIG-I initiate MAVS-mediated signaling leading to expression of type I IFNs. Specifically, the presence of sncRNAs or ERV-derived dsRNA in the cytoplasm are detected by RIG-I or MDA5, respectively, and initiate MAVS signaling post-IR treatments. Furthermore, AT-rich dsDNA released following irradiation may be transcribed by RNA polymerase III to produce cytosolic dsRNA which activates RIG-I–MAVS signaling. In this way, MAVS signaling plays a role in the production of type I IFNs following radiotherapy, although the precise contribution to overall antitumor immune responses remains to be determined. Abbreviations: double-stranded DNA (dsDNA); double-stranded RNA (dsRNA); endogenous retrovirus (ERV); interferon (IFN); ionizing radiation (IR); small non-coding RNAs (sncRNAs).

MAVS signaling has recently been implicated in type I IFN production after irradiation of cancer cells (Figure 2). Following treatment with IR, cytoplasmic small non-coding RNAs (sncRNAs) activated retinoic acid-inducible gene I (RIG-I; a cytosolic RNA sensor upstream of MAVS), initiating MAVS-mediated induction of type I IFNs [96]. Feng et al. (2020) demonstrated that MAVS and STING are both important for type I IFN production following IR, but the precise contribution of each pathway is dependent on the cell line being examined [97]. The authors propose that AT-rich dsDNA fragments released post-irradiation may be transcribed by RNA polymerase III at varying efficiencies depending on the cell line, providing the cytosolic RNA-ligand necessary to stimulate MAVS-regulated type I IFN production [97]. Previous studies are consistent with this notion, having shown that AT-rich dsDNA serves as a template for RNA polymerase III synthesis of dsRNA, which then activates RIG-I and subsequent MAVS-mediated production of type I IFNs [98,99]. Similar to RIG-I, melanoma differentiation-associated protein 5 (MDA5) is a cytoplasmic RNA sensor and upstream regulator of MAVS signaling. IR increased levels of cytosolic dsRNA derived from endogenous retrovirus activation, which induced interferon-stimulated genes through MDA5-MAVS signaling [100]. In summary, the exact contribution of MAVS- and/or STING-mediated type I IFN production post-IR treatment remains to be elucidated, however both pathways may play a role in inducing type I IFNs following radiotherapy.

## 6. Pathways Hindering Radiation-Induced cGAS-STING-Type I Interferon Signaling and Subsequent Antitumor Immunity

**Figure 3:**
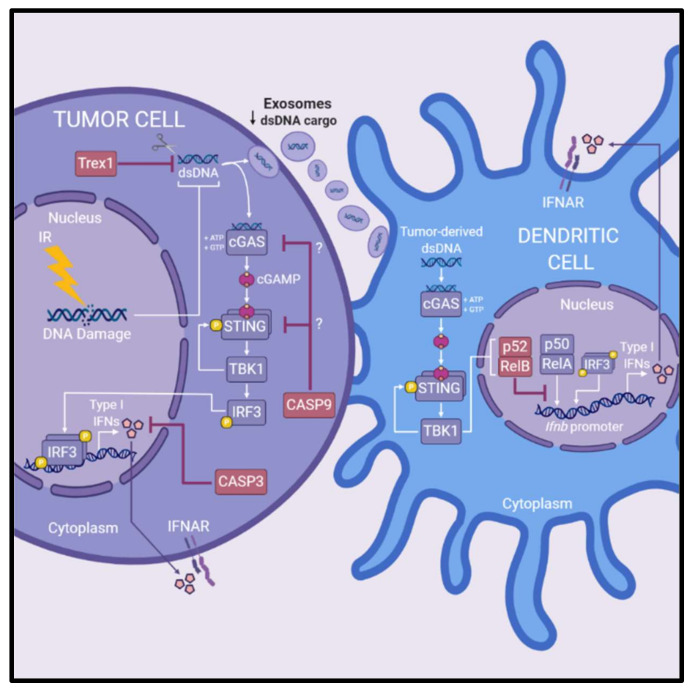
Pathways antagonizing radiation-induced cGAS-STING-type I interferon signaling. Several pathways hinder radiation-induced production of type I IFNs via cGAS–STING signaling and downstream antitumor immune responses. Trex1, a DNA exonuclease, degrades cytoplasmic DNA reducing the amount of ligand available for activation of cGAS–STING signaling in irradiated tumor cells. Induction of Trex1 in tumor cells following IR is also associated with reducing dsDNA cargo within exosomes, reducing the amount of substrate available for DC-mediated production of type I IFNs. Caspase 3 and caspase 9 are associated with reducing the production of type I IFNs of irradiated cancer cells and blunting the efficacy of IR in preclinical models. Caspase 3 is suggested to reduce production of type I IFN by facilitating cellular breakdown of irradiated cells. Caspase 9 is implicated in suppression of radiation-induced cGAS-STING signaling, however the exact mechanisms at play are not yet elucidated. Lastly, the efficacy of IR is reduced by non-canonical NF-κB signaling (involving the p52/RelB NF-κB complex) within DCs. Non-canonical NF-κB signaling inhibits RelA binding to the *Ifnb* promoter in DCs to regulate expression of type I IFN. Abbreviations: dendritic cells (DCs); double-stranded DNA (dsDNA); interferon (IFN); IFN-α/β-receptor (IFNAR); ionizing radiation (IR).

Several pathways have been identified that antagonize radiation-induced type I IFN production, thus attenuating optimal antitumor immune-mediated efficacy of IR (Figure 3). One antagonistic pathway is the caspase cascade that upon activation leads to a form of regulated cell death called apoptosis. Death of malignant cells due to apoptosis is considered a desirable outcome following exposure to various cytotoxic agents, including IR. Recent data oppose this view, as apoptotic caspases have been implicated in dampening STING-mediated innate immune signaling [84]. This unappreciated regulatory role of caspases was associated with resistance to radiotherapy in studies reported by Rodriguez-Ruiz et al. (2019) and Han et al. (2020). In the absence of caspase 3, IR enhanced IFNβ production of TSA breast cancer cells in vitro, and increased TSA tumor control in vivo, suggest that caspase 3 suppresses innate immune activation [101]. The authors propose that a deficiency of caspase 3 delays the breakdown of cells enabling greater secretion of type I IFNs [101]. Following IR treatment, caspase 9 was identified as a potent inhibitor of type I IFN production by tumor cells, which reduced succeeding antitumor T cell responses and the overall efficacy of IR [22]. In MC38 colorectal cancer models, IR was ineffective in controlling caspase-9-*proficient* tumors, but effective in controlling caspase-9-*deficient* tumors, except when either cGAS or STING were knocked-out simultaneously with caspase 9 [22]. This suggests that caspase 9 suppresses tumor-intrinsic DNA sensing involving cGAS–STING. These studies highlight the antagonistic role caspases may play in regulating innate immune responses following radiotherapy and thus hindering downstream immunological destruction of tumor cells, though the precise mechanisms remain to be elucidated.

Non-canonical NF-κB signaling within DCs has been implicated in restricting the efficacy of radiotherapy [102]. The non-canonical NF-κB pathway involves nuclear translocation of the p52/RelB complex to mediate gene expression, whereas the canonical NF-κB pathway involves p50/RelA [103]. Irradiated tumor cells triggered STING-dependent non-canonical NF-κB signaling in DCs which hampered optimal CD8+ T cell-mediated antitumor responses in preclinical MC38 colorectal cancer models [102]. Post-IR treatment, deficiency of non-canonical NF-κB in mice promoted adaptive antitumor immune responses by enhancing DC cross-priming capabilities and IFNβ production, indicating non-canonical NF-κB signaling negatively regulates DC function and subsequent radiation-induced antitumor immunity [102]. Further, inhibition of non-canonical NF-κB potentiated the efficacy of IR in vivo, suggesting targeting of this pathway could enhance radiotherapeutic outcomes [102]. Mechanistically, the authors show that non-canonical NF-κB signaling inhibited RelA binding to the *Ifnb* promoter in DCs to regulate IFNβ expression [102].

Trex1 is a DNA exonuclease that degrades cytoplasmic DNA thereby destroying the ligand responsible for radiation-induced activation of type I IFNs via cGAS–STING and subsequent antitumor immune responses [25]. The authors demonstrated that overexpression of Trex1 reduced therapeutic efficacy of IR in concert with immune checkpoint blockade in TSA breast cancer models [25]. Higher single doses of IR (12–18 Gy) induced Trex1 expression (correlating with decreased cytosolic DNA) in multiple breast cancer cell lines whereas lower single doses or multiple lower doses did not, indicating that single or multiple doses close to but below thresholds for Trex1 activation, may maximize type I IFN production following IR [25]. Intriguingly, induction of Trex1 decreased dsDNA within exosomes derived from irradiated cancer cells, abolishing the activation of type I IFN production by DCs [27]. Together, these findings suggest that increased levels of Trex1 not only reduce cytosolic dsDNA within cancer cells to limit type I IFN production, but also Trex1 restricts the capacity for exosomes derived from irradiated cancer cells to activate type I IFN production in DCs.

Altogether, these studies highlight avenues to potentiate radiation-induced innate immune activation (Table 3). Blocking caspase 3/9 activity, non-canonical NF-κB signaling, or Trex1 may be viable strategies for enhancing adaptive immune responses following radiotherapy, enabling better therapeutic outcomes.

## 7. Detrimental Effects of cGAS, STING, and Type I Interferons Following Radiation

Herein, we have discussed many mechanisms by which radiation-induced activation of the cGAS-STING-type I IFN cascade enhances therapeutic efficacy. However, it is important to note that these signaling pathways are multifaceted, having also been associated with driving resistance of tumors to CD8+ T cell-mediated killing, as well as other undesirable therapeutic outcomes.

Although type I IFNs are important components for driving antitumor immune responses, they paradoxically can protect cancer cells from immune-mediated killing. Tumor models established using IFNAR1-deficient tumor cells (i.e., MC38 colorectal carcinoma, B16F10 melanoma, and KPC pancreatic cancer cells) exhibited improved therapeutic responses to IR in a CD8+ T cell-mediated manner [104]. The authors further demonstrate that the improved response was due to reduced type I IFN-mediated induction of a granzyme inhibitor, Serpinb9 [104], thus making IFNAR1-deficient tumor cells more sensitive to CD8+ T cell killing. Furthermore, induction of type I IFN signaling post-IR has been shown to increase expression of the immunosuppressive indoleamine 2,3 dioxygenase 1 (IDO1) protein in cancer cells [105]. Inhibition of IDO1 improved efficacy of IR in CT26 and MC38 colorectal cancer models and was associated with an increased ratio of CD8+ T cells to immunosuppressive regulatory T cells [105]. Additionally, it has been shown that radiation as well as type I IFNs induce expression of the immune checkpoint ligand PD-L1 [106,107], and the STING pathway is likely involved (reviewed in [108]). PD-L1 binding to its receptor on CD8+ T cells dampens T cell cytolytic activity, however, clinically approved immune checkpoint inhibitors can potentially prevent this downmodulation. A combined radioimmunotherapy approach is supported by numerous studies that demonstrate synergy between radiation and anti-PD-1:PD-L1 immune checkpoint inhibition ([109,110,111] and reviewed in [112]).

Collectively, these studies provide insight into opposing antitumor effects regulated by radiation-induced type I IFNs.

Radiation-induced STING signaling reportedly contributes to immunosuppression in the TME, promotion of metastasis, and driving innate immune pathology. Irradiated MC38 colorectal cancer models recruited monocytic myeloid derived suppressor cells (MDSCs) to the TME in a STING-mediated manner which suppressed T cell function, reducing IR sensitivity [113]. Promotion of metastasis has been associated with cGAS–STING-mediated inflammatory responses [114] including in the context of radiotherapy [115]. Local IR treatment of 4T1 breast cancer models increased lung metastases in comparison to unirradiated groups [115]. Following IR, cGAS–STING activation in mesenchymal stem cells drove production of the chemokine CCL5. CCL5 enabled recruitment of macrophages that were essential for the observed increase in lung metastases [115]. Furthermore, DNA released from hepatocytes following irradiation of normal liver tissue triggered cGAS–STING-mediated signaling and type I IFN production in non-parenchymal cells (i.e., cells within the liver that are not hepatocytes), exacerbating liver injury [116].

Lastly, cGAS–STING signaling is involved in promoting a senescent cellular phenotype after exposure to IR (among other DNA damaging agents) [21,117]. Cellular senescence may be regarded as a desired cancer therapeutic outcome as mitotic progression ceases, however, several studies report the generation of therapy-induced senescent polyploid giant cells undergoing mitotic slippage that are capable of re-entering the cell cycle (i.e., escaping senescence) after prolonged periods of time following exposure to DNA damaging agents [54,82,83]. These “escaped” senescent polyploid giant cells have been proposed to contribute to genotoxic resistance, metastasis, and cancer recurrence following antitumor therapies [83,118,119], indicating that targeting of this cell population may be required for complete eradication of disease.

These data indicate that the use of IR to engage cGAS-STING-type I IFN signaling is nuanced and may also produce deleterious effects including radioresistance via immunosuppression, facilitation of metastasis, and inflammatory-driven pathology in normal tissue.

## 8. Augmenting STING Signaling to Enhance Radiation-Induced Antitumor Immunity

There is a plethora of STING agonists/activators being explored in combination with radiotherapy with the intention of potentiating antitumor immunity. Intratumoral administration of exogenous cGAMP to MC38 colorectal cancer models with local IR potentiated therapeutic efficacy and enhanced tumor-specific CD8+ T cells in a STING-dependent manner [26]. Similarly, inhalable phosphatidylserine liposomes loaded with cGAMP synergized with IR, better controlling metastases than either monotherapies, even outside of the irradiated area in B16-OVA and 4T1 lung metastases models [90]. A STING-activating nanovaccine (i.e., PC7A nanoparticle loaded with antigen) synergized with IR in TC-1 tumor models better controlling local irradiated tumors and distal unirradiated tumors than either treatment alone [120]. Lastly, RR-CDG (a STING agonist) synergized with IR in Panc02 pancreatic cancer models, producing robust CD8+ T cell responses that exerted control of both local and distant tumors [121]. These studies indicate that compounds directly activating STING may improve the antitumor efficacy of radiotherapy.

Other studies have observed enhanced type I IFN production, activation of cGAS–STING signaling, or increased antitumor immune responses using IR in concert with several other therapies. Chk1/2 inhibitors in combination with IR increased micronuclei formation (potentially increasing cGAS–STING activation) and type I IFNβ production in vitro and demonstrated improved immune-mediated tumor control in B16F10 melanoma models [80]. Hafnium oxide nanoparticles (i.e., NBTXR3) in combination with IR enhanced DNA damage in a colorectal cancer cell line leading to increased activation of the cGAS–STING pathway [122]. ATR inhibition potentiated type I IFN production in concert with IR in a cGAS–STING-dependent manner [97]. Moreover, ATR inhibition in combination with IR and immune checkpoint blockade resulted in superior antitumor efficacy in hepatocellular carcinoma models in a cGAS–STING-mediated manner [123]. As mentioned previously, one of the drawbacks of radiation-induced activation of the STING pathway is recruitment of immunosuppressive MDSCs to the TME [113]. A triple-modality strategy using IR, cGAMP, and anti-CCR2 (for depletion of MDSCs) displayed superior efficacy relative to mono- and dual-therapies indicating that removing MDSCs from the TME can further enhance radiation-induced antitumor immunity [113]. Overall, modalities that activate the cGAS-STING-type I IFN axis or preclude STING-mediated immunosuppression may represent solid candidates for enhancing the antitumor efficacy of IR.

## 9. Conclusions

IR is a crucial modality for treating a broad range of cancers and there is a growing appreciation for its capacity to harness antitumor immune activation. Here, we have reported on how IR activates an important nucleic acid sensing system, the cGAS–STING axis, to initiate early innate immune signaling that shapes subsequent adaptive antitumor immune responses. Although the cGAS–STING pathway likely evolved to counter pathogenic threats, radiotherapy can exploit activation of this pathway to bolster antitumor immunity. The potent DNA damaging capacity of IR leads to activation of cGAS–STING and subsequent type I IFN production within tumor cells or DCs, facilitating optimal stimulation of CD8+ T cell-mediated tumor destruction. However, the consequences of radiation-induced cGAS–STING signaling are nuanced and undesirable effects such as increased metastasis, immunosuppression, or damage to normal tissues have been reported. Further investigation is warranted to uncover the totality of consequences that radiation-induced cGAS–STING activation produces when treating cancer. The role that other innate signaling pathways (i.e., MAVS-associated signaling) may play in generating antitumor immune responses following radiotherapy should be noted. The precise contribution of STING- and/or MAVS-mediated type I IFN production is yet to be determined and a more nuanced understanding of nucleic acid sensing following IR could highlight additional avenues to enhance antitumor immunity. Furthermore, a broader understanding of the components contributing to the activation or inhibition of cGAS–STING signaling opens opportunities for improved therapeutic interventions to harness antitumor immunity following IR. Indeed, early investigations manipulating STING activation show promise for strengthening the therapeutic efficacy of radiotherapy.

## Figures and Tables

**Table 1:** Studies demonstrating cGAS–STING-dependent innate immune signaling following ionizing radiation.

Ref	Radiation Dose	Cells/Model	Cell/Tissue Type	Response(s)
[19]	1 Gy, 5 Gy ^b^	MEFs in vitro	mouse embryonic fibroblasts	micronuclei formation
[20]	20 Gy ^c^	MCF10A in vitro	human breast epithelial cells	micronuclei formation
[21]	12 Gy ^b^	MEFs in vitro; WI-38 in vitro	mouse embryonic fibroblasts; human lung fibroblasts	CCF ^a^ production
[22]	40 Gy	MC38 in vitro	mouse colon carcinoma	mtDNA ^a^ release into cytosol
[23]	75 Gy ^b^ in vitro pre-treatment	EG7 in vivo (s.c.) ^a^	mouse lymphoma	oxidized mtDNA
[24]	4 Gy whole mouse	C57Bl/6	liver	↓ ^d^ inflammatory factors with host STING-deficiency
[25]	8 Gy ^b^ × 3 ^a^	TSA and 4T1 in vitro MDA-MB-231 in vitro MCA38 in vitro	mouse mammary carcinomas human breast adenocarcinoma mouse colorectal carcinoma	↓ type I IFN ^a^ with cGAS- or STING-deficiency
[26]	20 Gy 40 Gy	MC38 in vivo (s.c.) MC38-SIY in vitro	mouse colon adenocarcinoma mouse colon adenocarcinoma (expressing tumor-antigen)	↓ efficacy with host STING-deficiency ↓ DC ^a^ activation + T cell stimulation with cGAS- or STING-deficient DCs
[27]	8 Gy ^b^ × 3	TSA in vitro	mouse mammary carcinoma	↓ DC activation with STING-deficient DCs
[28]	20 Gy ^b^	CT26 in vivo (s.c.)	mouse colorectal carcinoma	↓ efficacy with host cGAS-deficiency
[29]	1 Gy, 5 Gy ^b^	BMDMs in vitro	bone-marrow-derived macrophages	↓ type I IFN with STING-deficiency
[30]	8 Gy ^b^	E0771 in vivo (orthotopic)	mouse breast carcinoma	↓ efficacy with cGAMP depletion or host STING-deficiency

^a^ Abbreviations: cytoplasmic chromatin fragments (CCFs); dendritic cell (DC); interferon (IFN); mitochondrial DNA (mtDNA); subcutaneous (s.c.); 3 doses (× 3). ^b^ External beam X-ray irradiator. ^c^ γ-irradiator. ^d^ Decreased.

**Table 2:** Studies showing STING-independent interferon activation post-ionizing radiation.

Ref	Radiation Dose	Cells/Model	Cell Type	Responses
[96]	3, 6, 9 Gy ^c^ 5 Gy × 6 ^a,b^	MEFs in vitro; D54 in vitro; HCT116 in vitro D54 in vivo (s.c.) ^a^; HCT116 in vivo (s.c.)	mouse embryonic fibroblasts; human glioblastoma; human colon carcinoma	↓ ^d^ type I IFN ^a^ with MAVS- or RIG-I-deficiency ↓ efficacy with cellular MAVS- or RIG-I-deficiency
[97]	20 Gy ^b^	MCF10A in vitro; MC38 in vitro; MDA-MB-468 in vitro; PANC-1 in vitro; HCC1937 in vitro	human breast epithelial cells; mouse colon adenocarcinoma; human breast adenocarcinoma; human pancreas carcinoma; human breast carcinoma	↓ type I IFN activation with MAVS-deficiency
[98]	8 Gy ^b^	A549 B16F10	human lung carcinoma mouse melanoma	↑ ^e^ cytosolic dsRNA ^a^ + ERV ^a^ activation ↑ ISGs ^a^

^a^ Abbreviations: double-stranded RNA (dsRNA); endogenous retrovirus (ERV); interferon (IFN); IFN stimulated genes (ISGs); subcutaneous (s.c.); 6 doses (× 6). ^b^ External beam X-ray irradiator. ^c^ γ-irradiator. ^d^ Decreased. ^e^ Increased.

**Table 3:** Studies of pathways hindering cGAS–STING activation of interferon signaling after ionizing radiation.

Ref	Radiation Dose	Cells/Model	Cell Type	Responses
[101]	20 Gy ^b^ 8 Gy ^b^	TSA in vivo (s.c.) ^a^ TSA in vitro	mouse mammary carcinoma	↑ ^e^ efficacy with cellular CASP3-deficiency ↑ type I IFN ^a^ with cellular CASP3-deficiency
[22]	40 Gy 15 Gy	MC38 in vitro MC38 in vivo (s.c.)	mouse colon adenocarcinoma	↑ type I IFN with CASP9-deficiency ↑ efficacy with CASP9-deficiency
[102]	20 Gy	MC38 in vivo (s.c)	mouse colon adenocarcinoma	↑ efficacy, ↑ type I IFN, and ↑ DC priming capacity with host non-canonical NF-κB-deficiency
[25]	8 Gy ^b^ × 3 ^a^ + αCTLA4 8 Gy ^b^ × 3	TSA in vivo (s.c.) TSA in vitro	mouse mammary carcinoma	↓ ^d^ systemic (abscopal) efficacy with induction of cellular Trex1 ↓ type I IFN with induction of cellular Trex1
[27]	8 Gy ^b^ × 3	TSA in vitro	mouse mammary carcinoma	↓ dsDNA ^a^ cargo in exosomes with induction of cellular Trex1

^a^ Abbreviations: double-stranded DNA (dsDNA); interferon (IFN); subcutaneous (s.c.); 3 doses (× 3). ^b^ External beam X-ray irradiator. ^d^ Decreased. ^e^ Increased.

## Abbreviations

cDC1s	conventional dendritic cells type 1
CDN	cyclic dinucleotide
cGAMP	cyclic GMP-AMP
cGAS	cGAMP synthase
CCFs	cytoplasmic chromatin fragments
DAMPs	damage-associated molecular patterns
DCs	dendritic cells
dsDNA	double-stranded DNA
IDO1	indoleamine 2,3 dioxygenase 1
IFN	interferon
IFNAR	IFNα/β receptor
IRF3	interferon regulatory factor 3
IR	ionizing radiation
MHC I	major histocompatibility complex class I molecules
MDA5	melanoma differentiation-associated protein 5
MAVS	mitochondrial antiviral-signaling protein
mtDNA	mitochondria DNA
MDSCs	myeloid derived suppressor cells
RIG-I	retinoic acid-inducible gene I
STING	stimulator of interferon genes
TBK1	TANK-binding kinase 1
TCR	T cell receptor
TME	Tumor microenvironment
